# Fluid Cartilage as New Autologous Biomaterial in the Treatment of Minor Nose Defects: Clinical and Microscopic Difference Amongst Diced, Crushed, and Fluid Cartilage

**DOI:** 10.3390/ma12071062

**Published:** 2019-03-31

**Authors:** Angelo Trivisonno, Steven R. Cohen, Guy Magalon, Jèrèmy Magalon, Aris Sterodimas, Michele Pascali, Valerio Cervelli, Gabriele Toietta, Alfredo Colaprietra, Filippo Calcagni, Augusto Orlandi, Maria Giovanni Scioli, Pietro Gentile

**Affiliations:** 1 Department of Surgical Science University of Rome “La Sapienza”, 00161 Rome, Italy; angelo.trivisonno@uniroma1.it; 2 FACES+ Plastic Surgery, Skin and Laser Center, La Jolla CA 92121, USA and Division of Plastic Surgery, University of California San Diego, San Diego, CA 92121, USA; scohen@facesplus.com; 3 Cell Therapy Laboratory, CBT-1409, INSERM, Assistance Publique Hôpitaux de Marseille, 13005 Marseille, France; g.magalon@gmail.com; 4 Plastic Surgery Department, Assistance Publique Hôpitaux de Marseille (APHM), Aix Marseille University, 13005 Marseille, France; Jeremy.MAGALON@ap-hm.fr; 5 Department of Plastic Surgery, IASO General Hospital, Athens 15562, Greece; aris@sterodimas.com; 6 Department of Plastic and Reconstructive Surgery, University of Rome Tor Vergata, 00133 Rome, Italy; michelepascali.plasticsurgeon@gmail.com (M.P.); valeriocervelli@virgilio.it (V.C.); 7 Department of Research, Advanced Diagnostic, and Technological Innovation, Regina Elena National Cancer Institute, 00144 Rome, Italy; gabriele.toietta@ifo.gov.it; 8 Department of Plastic Surgery, Campus Bio-Medico University of Rome, 00128 Rome, Italy; colapietra.alfredo@gmail.com; 9 Department of Plastic Surgery Catholic University of the Sacred Heart, 00168 Rome, Italy; filippo.calcagni@gmail.com; 10 Department of Biomedicine and Prevention, Tor Vergata University of Rome, 00133 Rome, Italy; orlandi@uniroma2.it (A.O.); scioli@med.uniroma2.it (M.G.S.)

**Keywords:** fluid cartilage, minor nose defects, rhinoplasty, diced cartilage, crushing cartilage

## Abstract

Developing cartilage constructs with injectability, appropriate matrix composition, and persistent cartilaginous phenotype remains an enduring challenge in cartilage repair. Fourteen patients with minor contour deformity were treated with fluid cartilage filler gently injected as autologous fluid graft in deep planes of defect of the nose that were close to the bone or the cartilage. A computerized tomographic scan control was performed after 12 months. Pearson’s Chi-square test was used to investigate differences in cartilage density between native and newly formed cartilages. The endpoints were the possibility of using fluid cartilage as filler with aesthetic and functional improvement and versatility. Patients were followed up for two years. The constructs of fluid cartilage graft that were injected in the deep plane resulted in a persistent cartilage tissue with appropriate morphology, adequate central nutritional perfusion without central necrosis or ossification, and further augmented nasal dorsum without obvious contraction and deformation. This report demonstrated that fluid cartilage grafts are useful for cartilage regeneration in patients with outcomes of rhinoplasty, internal nasal valve collapse, and minor congenital nose aesthetics deformity.

## 1. Introduction

The rhinoplasty is mainly a procedure for the reduction of the size, but sometimes the need of enlargement for defects or irregularities, in primary or secondary rhinoplasties. Reconstructive rhinoplasty can be also performed to correct deformities following nasal cancer surgery. To rebuild the hard tissue defects of the nose, the first choice is the autologous cartilage. The hardest part is a careful remodelling of the cartilage grafts, especially in noses with thinner soft tissues, where the edges of the grafts cannot be camouflaged. So, it would be possible to obtain a good result using a more malleable crushed or diced cartilage in larger pieces [1], or in smaller (less than 0.2 mm) [2] wrapped or not in fascia, or Surgicel^®^ (oxidised regenerated cellulose-polyanhydroglucuronic acid, which is commonly used as haemostatic agent, www.ethicon.com), or acellular matrix. Sometimes to aggregate using fibrine glue or patient’s own blood, or to implant them, it is necessary to incise and dissect the nose tissues as classic rhinoplasty. To avoid these invasive procedures, especially when we do not need large quantities of an implant, an easier procedure is the use of more fluid cartilage derived by shaving with scalpel n. 15, which we can manipulate as a filler with injection by needles between 21–18 gauge, or equivalent size cannulas.

Autologous fluid cartilage obtained from the shaving procedure of the cartilage septum provides an autologous source of filler, and it has been demonstrated to be effective in the treatment of nose defects in the dorsum of the nose and alar cartilage defects, or the correction of sovratip imperfections; moreover, the incomparable biocompatibility enabled shaving cartilage to be a promising autologous biotechnology for tissue engineering and aesthetical purposes [3]. Other donor sites can be the rib cartilage and the chonca ear auricular cartilage, but with different peculiarities than those of the nasal cartilage.

Unfortunately, the poor mechanical stability and rapid degradability of the shaving cartilage leads to shrinking and deformed cartilage formation in vivo. Hence, the use of saline solution loaded with shaving fluid cartilage into the defect site is highly desirable for cartilage repair or for the correction of nose defects; in particular, the use of fluid cartilage represents a microinvasive procedure, and grafts are more flexible to fill the lesions [3].

In the current day and age, the primary issue when it comes to translating experimental tissue engineering protocols into routine clinical care revolves around identifying accessible sites with the right level of cartilage collection [4,5]. Furthermore, the requirement of specificity in relating to describing the technical procedure along with its safety is a critical factor. This is true for outcomes such as those of rhinoplasty or nose defects correction. Sometimes, this depends on the smaller dislocation of the spreader grafts, especially at the osseocartilaginous k-area.

The human body contains many niches or loci that contain a considerable amount of stem cells; however, they can often not be accessible easily and have high residual morbidity when it comes to the anatomical site [6,7,8]. The nasal septum is a niche that holds chondrocytes. It has both plasticity and multipotential capability. This is an easily accessible niche, one that holds a limited morbidity once micrografts have been collected. Ba et al. [9] in a previous study created what is known as the cell bricks technique. This technique involves culturing a chondrocyte sheet; an extracellular matrix (ECM) complex is cut into cell bricks (multiple small fragments). This work concluded that chondrocyte bricks considerably took up space within vascular infiltrations into PRP gels. This resulted in a slowing of their breakdown, and allowed the framework and the shape of the PRP grafts to be maintained [10]. The study put forth the hypothesis that the PRP gel that had been enriched by the cell brick could be the perfect niche to be injected into adult stem cells. This could in turn lead to a regeneration of the cartilage tissue through consistent cartilaginous phenotype, uniform histological structure, and a smaller level of degradation [10].

For this piece of research, we examined clinical results obtained by the use of fluid cartilage as filler, and also we analysed the in vivo performance of human chondrocytes (HCs) contained in fluid cartilage in cell brick-enriched saline solution evaluating the persistence of a stable chondrogenic phenotype.

There are two main techniques through which chondrocytes may be cultured. The first method involves collecting the cartilage tissue under sterile conditions and then putting it through an enzyme digestion process [6,11,12]. The resulting cell suspensions are added to a culture dish alongside a specific medium that has the needed additives. Then, the mixture is incubated. The colonies that are developed through this are further subcultured, right before confluence, and the cells go through stimulation so that they are able to differentiate. The second method is putting the cartilage through mechanical centrifugation [12]. This approach is innovative and helps isolate chondrocytes. Here, the cartilage is subjected to grafts with the assumption that it is similar to any other tissue that plays a connective role. Without manipulating the matrix, there is a collection phase, and then one phase during which the mechanical disaggregation of the tissue takes place.

We intended to clear up the clinical and microscopical impacts of fluid cartilage nose injection in people affected by outcomes of rhinoplasty or for aesthetical purpose in the treatment of minor contour deformity. The information we reported exhibits the clinical efficacy and histological safety of the fluid cartilage treatment. Additionally, patients’ fulfilment and computerised tomographic scan (CT) examination have affirmed the quality of the outcomes.

## 2. Materials and Methods

This observational case-series study was conducted following the principles outlined in the Declaration of Helsinki and internationally consented ethics in clinical research [13]. A quality assessment was carried out based on the Strengthening the Reporting of Observational studies in Epidemiology (STROBE) checklist [14]. The study protocol, which was the object of two university master’s degrees titled “Plastic aesthetic surgery of facial district” and “Regenerative surgery and medicine in wound care management”, was approved with Rectoral Degree (D.R. n. 1794/2018) of 19 September 2018 and the Ethics on Research Committee of the School of Medicine, “Tor Vergata” University, Rome, Italy, with registration number #0031036/2018. All of the patients received detailed oral and written information about the study, including the risks, benefits, and alternative therapies, and signed an informed consent form before any study procedures. The study protocol was performed following the European rules (1394/2007 EC), European Medicine Agency (EMA) and Committee for Advanced Therapies (CAT) EMA/CAT recommendations (20 June 2014 EMA/CAT/600280/2010 Rev 1).

### 2.1. Patient Population

A total of 14 patients, eight females and six males aged 24 to 71 years (Table 1), affected by minor contour irregularities as nose defects in primary and secondary rhinoplasty were treated with the injection of fluid shaving cartilage, evaluating the long-term results. The harvesting of cartilage was done from septal cartilage and the associated deviation of the nasal septum if any was corrected at the same time.

The material was injected with 21 gauge needles or blunted tip lipofilling cannulas with 1.2-mm internal diameters and 0.8-mm hole sizes. Success was defined as the long-term survival of the graft in the desired site and the absence of recurrent deformity or complications such as extrusion, infection, or displacement.

Three patients underwent nonsurgical primary rhinoplasty, two patients underwent secondary rhinoplasty, and nine patients underwent nonsurgical sequelae rhinoplasty. The most common indications were minor contour irregularities. It is very important for the authors that there was an incremental injection of the fluid cartilage to the desired location with repeated visual and tactile evaluation after each injection.

Fundamental and local prohibition criteria were considered. Fundamental expulsion criteria included immunosuppression, cancer, sepsis, and diabetes. Localised expulsion criteria included the utilisation of topical filler (fat graft, hyaluronic acid) and the nasal spray that is commonly used for nasal obstruction earlier in the year.

Diagnoses of minor contour irregularities and the selection of the patients were established performing:

- Detailed therapeutic history (the outcomes of rhinoplasty or outcomes of trauma);
- Clinical examination (alar nose defects, dorsum, radix, and supra tip with minor contour deformity, nasal valve collapse);
- Blood test and urinalysis;
- CT scans

### 2.2. Fluid Cartilage Procedure

#### 2.2.1. Fluid Cartilage Preparation

**Figure 1:**
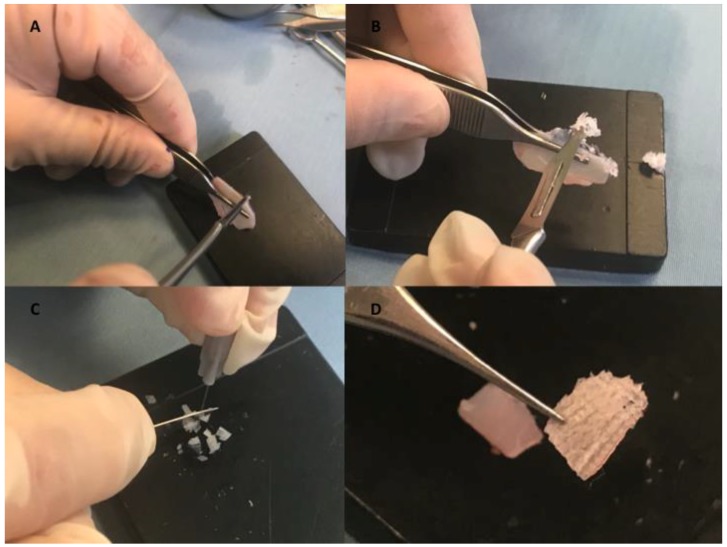
Autologous fluid shaving cartilage preparation compared with other products. (**A**) Cartilage preparation using as source nasal septum (with or without perichondrium). (**B**) Soft tissue attachments were debrided from the cartilage block using Adson Brown forceps in the non-dominant hand of the surgeon and surgical blade (number 15) in the perpendicular plane to scratch the edges of the cartilage block with very gentle pressure shaving very thin and small pieces. (**C**) Diced cartilage. (**D**) Crushing cartilage.

Autologous fluid shaving cartilage for immediate clinical filler was prepared using nasal septum (with or without perichondrium) as the source (Figure 1A). The procedure was performed in absolute sterility conditions and in an operating room during the operation, using two different surgical servant tables (one supporting surgical instruments for rhinoplasty and one for fluid cartilage preparation).

**Figure 2:**
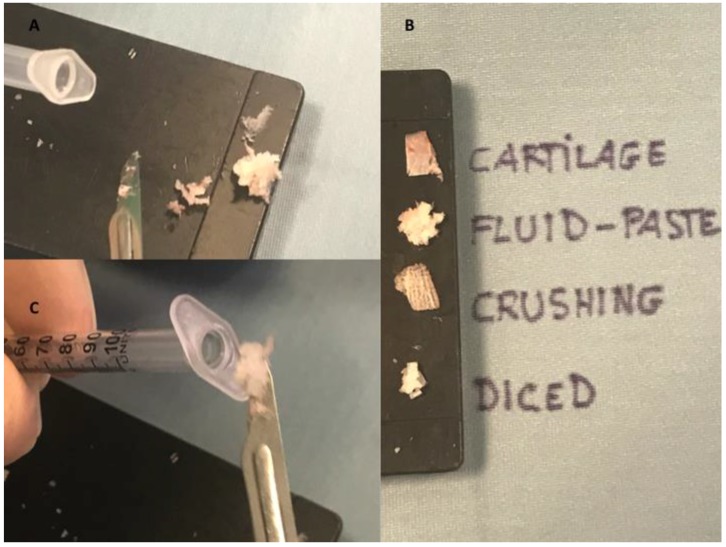
All cartilage products obtained. (**A**,**B**) Fluid, diced, and crushing cartilage products obtained by septal cartilage in comparison. (**C**) The insertion of cartilage paste obtained in a 1-mL Luer lock syringe.

For the preparation of the injectable shaving cartilage, any soft tissue attachments were debrided from the cartilage block. It was strongly held firm by Adson Brown forceps in the non-dominant hand of the surgeon; a surgical blade (number 15) was used in a perpendicular plane to scratch the edges of the cartilage block with very gently pressure shaving very thin and small pieces (Figure 1B). No visible separate pieces of cartilage could be identified, and the produced material rather resembled as a powder. Two harder sheets of cartilage that included a bigger and softer central part were observed (see Video S1). The authors compared during the procedure of different diced (Figure 1C) and crushed (Figure 1D) cartilage products. All of the cartilage products were reported in Figure 2A,B.

In the study only septal cartilage has been used, because it was considered more appropriate in comparison to rib and conchal cartilages, which was more respectively pliable, fragile, and not linear. Then, the material was soaked in sterile saline solution, because there was a dehydration of the cartilage during the shaving, which also has adhesive effects, but leaves its fluidity. Then, it was introduced to 1-ml Luer lock syringes (Figure 2C).

#### 2.2.2. Indications and Injection of Fluid Cartilage as Filler

The surface was put through a visual and tactile analysis to find the best site for injection. It was conducted as part of surgical and non-surgical rhinoplasty. This fluid graft was applicable for each section of the nose, including the dorsum, radix, nasal tip, premaxilla, alar base, columella, soft triangle, and more. In more detail, it was used to either: correct the pinch-nose deformity or the external nasal valve collapse; enhance the radix and soften the acute nasofrontal angle; soften the accentuated supra tip break; correct the depressions of the collapsed upper lateral cartilages if there were not breathing problems; or instead laterally correct the septum through the use of spreader grafts, when it is required to improve the breathing. Moreover, it could also be used to camouflage minor irregularities derived from previous traditional cartilage grafts or their displacement. It is possible to use it to increase the columellar labial angle by augmenting the premaxilla, restore enough volume to the dumplings around the piriform area, and restore a diverge appearance at the location of deficient footplates in the base of the columella.

The injection was performed at the deep level in contact with the cartilage or bone in closed space, without undermining the skin flap, to make sure that the graft is fixed in its place, and getting rid of any and all possibility that it may find itself displaced in the future. Vice versa, superficial layers were not considered because of the high risks of intravascular injection and the different kinds of tissues according to the like-to-like principles, and also for the difficulties of removing the graft if it is necessary.

The fluid graft must be manipulated as a filler, introduced very “gently” through 21-gauge needles or blunted tip lipofilling cannulas with 1.2-mm internal diameter but with an 0.8-mm hole size, and the process of injection was incremental and cautious (see video). The tactile and visual examination was repeated until the point at which the right amount had been injected. A gentle massage of the skin above the injected material could help with the final moulding; however, this would only be possible if the injection had not been directed into a tight pocket.

According to Ali Manafi et al. [3], if injected into the periorbital or dosral nasal area, the injected material could experience embolisation in the ophthalmic artery. The vasoconstrictor impact of adrenaline meant that this risk was minimal. The adrenaline had to be injected prior to the graft; the likelihood of embolisation would be much lower if sharp needles were avoided and a blunt-tip cannula was used instead.

#### 2.2.3. Clinical Observation

The rhinoplasty was preceded by standard photography, which recorded lateral, frontal, and ¾ basal views. This was done during follow-ups as well: one at six months, one after a year, and so on.

The plastic surgeons conducted a final analysis of the results through tactile and visual data. Some complications may present as a result of the operation, including extrusion, resorption, infection, and overcorrection—the follow-up inspections kept all of these problems in mind. The procedure was considered successful if no recurrent deformity presented itself, and if the graft was able to survive long term.

The patients were given a survey to gauge how happy they were with the rhinoplasty a year after they had undergone the procedure. They were able to fill out the questionnaire without revealing their identity. The questionnaire was collected into a single folder, which held responses from all of the patients that had undergone the procedure. Patients noted whether they were satisfied, unsatisfied, or very satisfied with the results.

#### 2.2.4. Histological Evaluation

Cartilage samples from the different procedures were collected in 10% neutral buffered formalin or physiological saline solution for histology and human chondrocyte isolation, respectively. Microscopic evaluation of routinary Haematoxylin–eosin-stained paraffin-embedded sections was performed to morphologically analyse cartilage tissue from different procedures [15].

#### 2.2.5. Chondrocyte Isolation and Culture

Human chondrocytes were freshly isolated, as reported [15]. Briefly, for enzymatic digestion, cartilage pieces were pretreated with 0.25% trypsin/1 mM EDTA for 45 min at 37 °C and then incubated with 0.15% type II collagenase (Worthington Biochemical Corporation, Lakewood, NJ, USA) in Dulbecco’s modified Eagle’s medium (DMEM, Sigma Aldrich, Saint Louis, MO, USA) for 22 h at 37 °C with shaking. Cell number calculated using a hemocytometer with trypan blue exclusion. HCs were seeded in plastic dishes and cultured in DMEM supplemented with 10% foetal bovine serum (FBS), 2 mM of L-glutamine, antibiotics (100 U/mL penicillin, 100 mg/mL streptomycin), and amphotericin B (0.25 mg/mL) at 37 °C in a humidified atmosphere with 5% CO_2_. After seven days in culture, chondrocytes were used for histochemical analysis and RNA extraction.

#### 2.2.6. Cytospin and Alcian–PAS Staining

HCs were made to adhere to a glass slide by cytospin [12], and then Alcian–PAS staining was performed (Ventana-Roche Diagnostics; Milan, Italy). Cells were analysed under a light microscope (Eclipse E600, Nikon, Tokyo, Japan) and microphotographs were captured by a DXM1200F Digital camera (Nikon) using ACT-1 software (Nikon).

#### 2.2.7. RT-PCR Analysis

Total RNA extraction from cultured HCs was performed using the TRIZOL reagent according to the manufacturer’s instructions (Invitrogen, Carlsbad, CA, USA). Reverse-transcriptase polymerase chain reaction (RT-PCR) was performed using Platinum^®^ Taq DNA Polymerase (RT-PCR, Invitrogen). The following primers for the main chondrogenic markers were used: collagen type II alpha 1 chain (COL2A1) sense 5′-CAACACTGCCAACGTCCAGAT-3′ and antisense 5′CTGCTTCGTCCAGATAGGCAAT-3′, aggrecan (ACAN) sense 5′-TACTCTGGGTTTTCGTGACTC-3′ and antisense 5′-CGATGCCTTTCACCACGACTT-3′, housekeeping gene GAPDH sense 5′-ACGGATTTGGTCGTATTGG-3′ and antisense 5′-GATTTTGGAGGGATCTCGC-3′ [16].

#### 2.2.8. Statistical Analysis

Values as the mean in addition to baseline mistake were examined by means of the Statistical Package for the Social Sciences (SPSS), version 19.0 (IBM; http://www-01.ibm.com). The normality of quantitative variables was tested by the Kolmogorov–Smirnov test. Human chondrocytes detected were expressed as mean plus or minus the standard deviation (SD).

## 3. Results

### 3.1. Histological, Morphological, and Phenotypic Analysis

**Figure 3:**
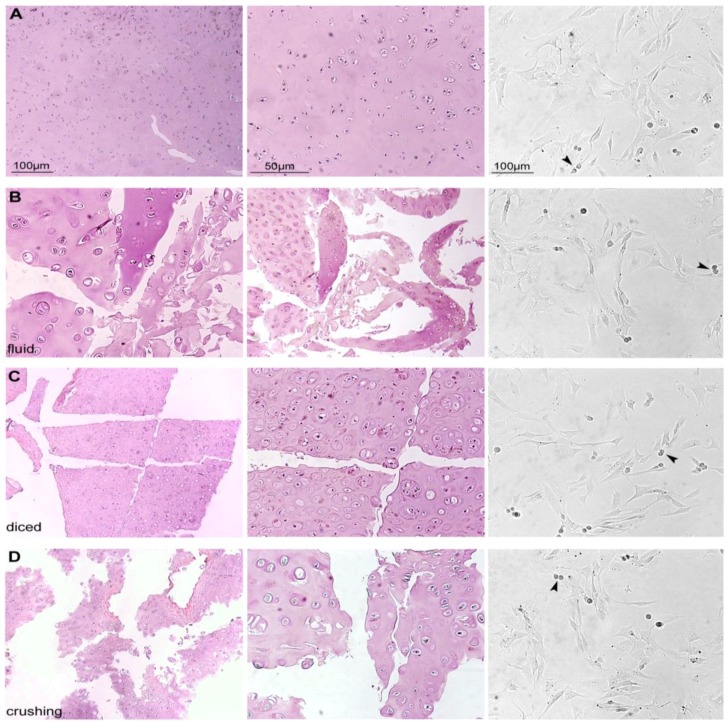
Histological and morphological analysis of cartilage tissue and cultured human chondrocytes (HCs) from different procedures. (**A**–**D**) Haematoxylin–eosin-stained paraffin-embedded sections of cartilage samples from the different procedures (left and center) and the corresponding cultured HCs (right); from top to bottom: whole cartilage, fluid, diced, and crushing samples. Arrow head, mitosis.

Haematoxylin–eosin-stained sections (Figure 3A–D) of cartilage samples obtained from the different procedures showed, in each group, the presence of connective tissue consisting of a dense matrix and HCs embedded. Although the different procedures induced an evident manipulation of the connective tissue, the overall structure of HCs appeared well conserved in all groups. In addition, isolated HCs (overall cell yield 120,000 ± 11,800) from these cartilage samples (~1 cm^2^) were alive (>99% by trypan blue exclusion) and able to adhere in plastic dishes as well as proliferate (Figure 3A–D), mitosis = harrow heads), showing the same morphological features characteristic of chondrocytes.

**Figure 4:**
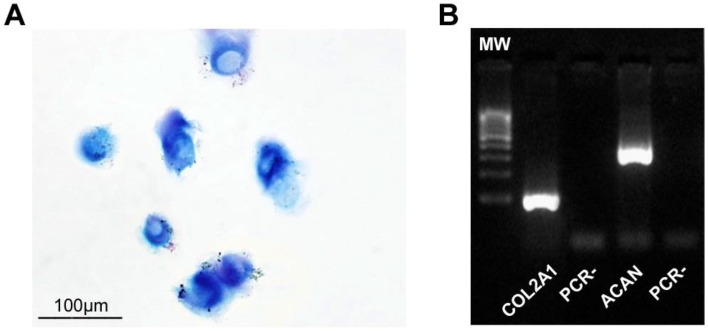
Phenotypic characterisation of cultured HCs. (**A**) Representative image of Alcian–PAS staining of cytospinned HCs (in particular, fluid sample). (**B**) Representative RT-PCR showing chondrogenic marker COL2A1 and ACAN mRNA expression (in particular, fluid sample). Abbreviations: MW, molecular weight; PCR-, negative control.

Moreover, cultured HCs, from all procedures, still produced glycosaminoglycans (GAGs), as shown by Alcian–PAS staining (Figure 4A), and expressed specific chondrogenic markers such as COL1A2 and ACAN (Figure 4B).

### 3.2. Clinical Long-Term Results and Instrumental Observation

**Figure 5:**
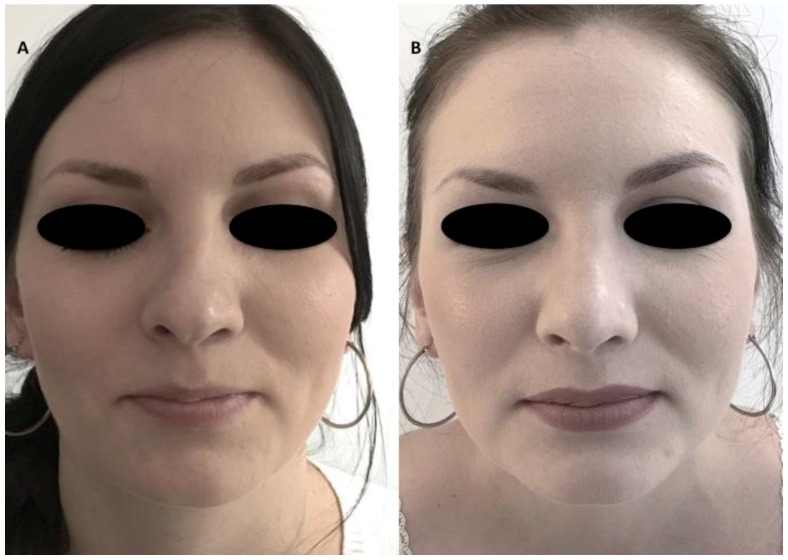
43-year-old female patient affected by nasal obstruction and minor soft tissue defects in frontal view. (**A**) External nose analysis showed external nasal valve collapse, deficit of supra tip projection, deficit of radix and dorsum volume. Anterior rhinoscopy showed a deviated nasal septum and bilateral stenosis of the internal valves. (**B**) The fluid cartilage as filler was injected on the external nasal valve collapse, in the alar cartilage side, or in the radix, or in the nasal dorsum or in the supra tip area. Post-operative follow-up evaluation, 12 months after the fluid cartilage injection showed optimal aesthetic results and an improvement of nasal obstruction.

**Figure 6:**
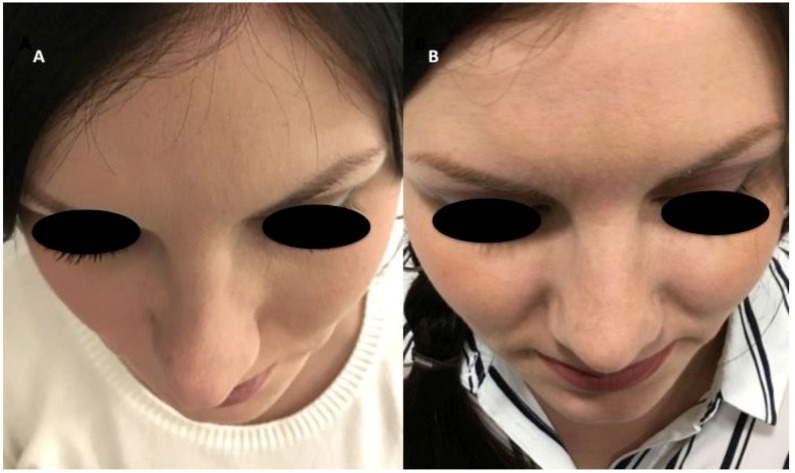
43-year-old female patient affected by nasal obstruction and minor soft tissue defects in detail projection. (**A**) External nose analysis showed better-left nose deviation, external nasal valve collapse, deficit of supra tip projection, deficit of radix and dorsum volume. Anterior rhinoscopy showed a deviated nasal septum and the bilateral stenosis of the internal valves. (**B**) The fluid cartilage as filler was injected on the external nasal valve collapse, in the alar cartilage side, in the radix, in the nasal dorsum, or in the supra tip area. Post-operative follow-up evaluation, 12 months after the fluid cartilage injection, showed optimal aesthetic results and an improvement of nasal obstruction.

The authors reported the results obtained using autologous fluid cartilage as filler. Nasal obstruction was identified in these patients; external nose analysis showed external nasal valve collapse, a deficit of supra tip projection, and a deficit of radix and dorsum volume (Figure 5A and Figure 6A). Anterior rhinoscopy showed a deviated nasal septum and bilateral stenosis of the internal valves. The fluid cartilage as filler was injected on the external nasal valve collapse, in the alar cartilage side, in the radix, in the nasal dorsum, or in the supra tip area, depending on the defect of the patient. Post-operative follow-up evaluation has shown optimal aesthetic results and the improvement of nasal obstruction. In particular, the authors evaluated this improvement through the Nasal Obstruction Symptom Evaluation (NOSE) survey [17]. The NOSE survey is a validated specific instrument designed to measure nasal obstruction that is commonly used in otolaryngology practices to provide an objective measure of nasal obstruction [17]. It is a brief questionnaire consisting of five self-rated items, each scored from 0 to 4. The NOSE score represents the sum of the responses to the five individual items and ranges from 0 to 20. The mean (SD) NOSE score was 8.2 (6.1) (range, 0 to 20).

This autologous filler provides functional supports to the alar cartilages, which are usually collapsed because of excessive resection during previous surgery, or improves the volume in the dorsum, which is in the radix.

**Figure 7:**
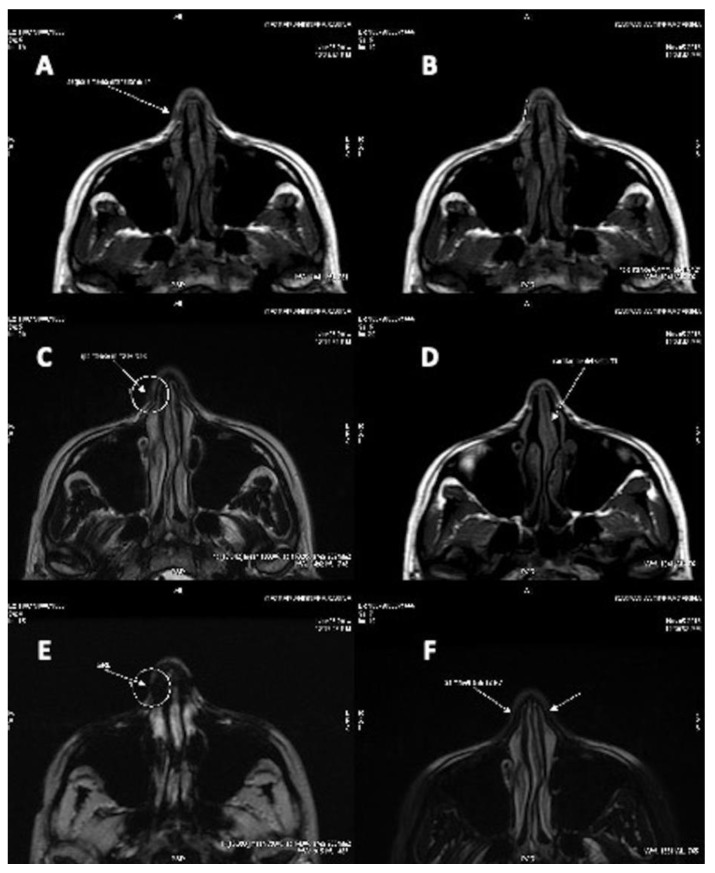
Computerised tomographic (CT) scans of patient showed in Figure 4. (**A**,**C**,**E**) Pre-operative situation. (**A**) CT scans, T1 medium signal intensity, showed the pre-operative situation with soft tissue defect of the nasal tip and nasal septum deviation. (**B**) CT scans, T1 medium signal intensity, showed the post-operative situation with an improvement of soft tissue volume in the treated area. (**C**) CT scans, T2 high signal intensity, showed the pre-operative situation in detail, with cartilage defect and nasal valve collapse. (**D**) CT scans, T2 high signal intensity, showed the post-operative situation with correction of the nasal valve collapse. (**E**) CT scans, T2 high signal intensity, showed the detail of the pre-operative situation with soft tissue defect of the nasal tip and cartilage defect in detail. (**F**) CT scans, T2 high signal intensity, of the same area after 12 months show the regenerated site in the post-operative image with soft tissue volume improvement and the correction of the nasal septum deviation.

The questionnaire was given to the 14 patients at one year or more after the operation, and general satisfaction was evaluated. From those, seven patients (50% ± 5.0%) marked very satisfied, four patients (30% ± 2.5%) marked satisfied, and three patients (20% ± 2.0%) marked that they were unhappy with the results. No cases of infection, extrusion, displacement, or excess bulging occurred during the course of follow-up. None of the patients experienced complete resorption and the recurrence of pre-existing deformity, but partial resorption was seen in almost all of the patients to small extents. Resorption evaluation was estimated by CT scans analysed by a radiologist comparing pre-operative (arrows in Figure 7A,C,E) and post-operative situations (arrows in Figure 7D,F). Clinical results were shown in Figure 5B and Figure 6B. In the CT scans, the pre-operative situation (Figure 7A,C,E) was shown in comparison with the regenerated site in the post-operative image (Figure 7B,D) with correction of the nasal valve collapse. In particular, CT scans performed after 12 months (Figure 7F) showed an improvement in soft tissue volume and the correction of the nasal septum deviation.

## 4. Discussion

Rhinoplasty is the technique through which the superior airways are corrected through an anatomical and functional alteration. Conventional techniques to treat major irregularities of the nose include the use of cartilage graft as “spreader grafts”. These help with functional support to the lateral cartilages’ lower portion. These would normally collapse due to the excessive resection caused by prior surgeries, or because of nasal bone detachment which is a result of the surgery, providing support to the internal nasal valve [18]. Non-absorbable sutures are used to fix and implant them. The sutures are able to perfectly stabilise the graft, because they aren’t exposed at the level of the surface. In terms of pure biology, the graft sustains itself and finds nourishment through imbibition. This is because it has created a fresh microvascular bridge between the receiving bed and the graft. This effectively means that if the graft is unstable, irroration may not find a perfect balance, and this may lead to reabsorption and dehydration overtime.

On the other hand, minor contour irregularities of the nasal surface produced by outcomes of rhinoplasty or trauma could be seen as trifle; however, they’re at times the only elements keeping us from the perfect result [3]. In many cases, at the end of the procedure, some irregularities remain—even after the main adjustments for the osseocartilaginous skeleton have been completed. Using common grafting material to address these irregularities can be difficult at times. Cartilage blocks, crushed cartilages, and fascia come with their own set of issues. Blocks lead to visible, palpable edges. Crushed versions can be unpredictable when it comes to resorption, and autologous soft tissue materials or fascia require an altogether different site for harvest [3].

Another problem that is normally not considered is that malleable materials, when used in open rhinoplasties without suture fixation, can result in displacement once the skin flap returns to its original position. How effective the graft is when it comes to completely resolving contour deformity can’t be examined till the skin is put place in its place. Therefore, the graft may need to be repeatedly revaluated. Each time, the risk of the skin’s movement will grow; this is the reason that it is better for the implantation to be conducted in a closed area.

Regenerative plastic surgery and tissue engineering open new challenges for rhinoplasty and the treatment of irregularities of the nasal surface. In the context of cartilage regeneration, creating an injectable approach could link with the demand for microinvasive surgery [19].

Unlike solid scaffold-based tissue engineering, constructs for injectable cartilage need fluidity and the immediate development of a chondrogenic niche [10]. This proves that fragmented chondrocyte macroaggregates (cell bricks) have the ability to stabilise PRP gel effectively in vivo and aid chondrogenesis with stable morphology. The research shows that an injectable complex could help chondrogenesis of bone marrow mesenchymal stem cells (BM-MSCs) in vivo, and showed that a biological graft that was this complete can meet nasal augmentation requirements, and therefore hold future promise in craniofacial reconstruction [8]. The study’s most significant finding is that BM-MSCs within chondrocyte brick (CB)-enriched PRP gel underwent persistent chondrogenesis, and hypertrophic translation was prevented [10]. Moreover, BM-MSCs have the ability to push angiogenesis via Growth Factors (GF) secretion, in specific, Vascular Endothelial Growth Factors (VEGF) [20].

Angiogenesis is an important factor when it comes to the regeneration of tissues [21]. This finding “warranted reduction of the donor cartilage compared with conventional chondrocyte transplantation and provides a microinvasive approach for cartilage regeneration, indicating a promising use of cell bricks in clinical application in the future. For clinical translation, donor cartilage could be harvested from the nasal septum”.

One important upside of using PRP gel is that it can result in tissue modelling, which is faster than synthetic polymers. It can push the wound to heal. A study by Ba et al. [9] presented a surprising phenomenon whereby the BM-MSCs combined with CB-PRP gel resulted in angiogenesis in BM-MSC regions and hindered the graft’s central necrosis. During their own past experiment, the CB-PRP mixed with chondrocytes lead to a necrosis of the constructs’ interior. This could be the result of the chondrocytes robust anti-angiogenic capacity. The study showed that a higher VEGF expression was found when it comes to BM-MSCs in CB-PRP gels. This was thought to help angiogenesis in BM-MSCs within PRP grafts [9].

Actually, in 2019, according to the Italian rules and EMA/CAT recommendations, it is not more possible to mix PRP with BM-MSCs or other cellular material. For this reason, the authors did not consider the possibility of using PRP as a scaffold for fluid cartilage.

Lately, nonsurgical rhinoplasty that uses various filler materials has become popular. Removing the requirement for anaesthesia or an operating table, the procedure makes early and adjustable improvement possible. Even safer fillers such as hyaluronic acid can cause complications in some rare cases [22]. However, it is their short-term durability that is the real drawback [23,24]. Furthermore, permanent fillers are used in a limited fashion because of their possible side effects, which include extrusion, infection, and reaction [25].

Injectable autologous material, which can be implemented underneath the nasal skin, can be useful in terms of ideal surgery results. The best donor site is the nasal septum that is hyaline cartilage such as the cartilage framework of the nose, with or without perichondrium. The second choice is the rib, because it is hyaline cartilage too, with a large quantity available; however, it needs a more invasive harvesting procedure. We have used the chonca ear cartilage in two cases, but then it has been discarded because it is inadequate for volume considering thin thickness and quality; in fact, it is elastic cartilage, which is different from the nasal framework, and is difficult to shave.

Several manipulations make the cartilage more manageable when it comes to camouflage; the reason for this is that the shape and consistency is heavily reliant on the harvest location. Diced, minced, and crushed cartilage used with or without fascia wrapping are notable examples [3].

Many different processes are used to crush it into the needed consistency. This includes a cottle cartilage crusher and morseliser. It can be moulded in an easier manner with fewer palpable edges underneath the skin. This is not time consuming, and can be easily prepared. However, the main issue is that the crushing itself results in damaging the lacuna’s structure, and can result in reducing the chondrocytes’ viability [26].

Cackmack [26] has outlined a classification for the level of crushing that is feasible. This work analysed the longer-term permanence along with the viability of every class found in experimental work. This study shows that that the more cartilage that is crushed, the lesser the likelihood of it surviving when used as a graft [27].

When a surgeon crushes a cartilage to the point that it is injectable, there is a higher chance that the deformity will reoccur and further resorption will take place [27].

Peers described this process many years ago [28]; however, the discussion had not pertained to rhinoplasty. Several studies outlined diced cartilage since then; however, the method has never been employed in large numbers. However, during the last few years, the method has started to pique interest, and its use to enhance rhinoplasty has thus resurfaced. The described diced cartilage was used in larger pieces [1] but also in smaller pieces (less than 0.2 mm) [2] wrapped or not in fascia, Surgicel (oxidised cellulose polymer), or acellular dermal matrix to obtain a smooth consistency. Sometimes, fibrin glue or the patient’s own blood was used to aggregate the diced cartilage. However, to implant them, it is necessary to incise and dissect the soft nose tissues, such as in classic rhinoplasty.

Surgeons will typically opt to use diced cartilage instead of a solid piece because it allows for more flexibility and comes with a much lower risk of warping. Furthermore, it obviates the need for a single large graft. Extrusion or absorption is a key problem for biomaterial, and the same is noted as an issue when it comes to diced cartilage rhinoplasty [29].

Surgeons have employed alloplastic, synthetic, or autogenous wraps to work around issues such as the visibility of the grafts or palpability. The former allow for the cartilage construct to remain in camouflage. A scaffold to deliver diced cartilage has not yet been determined; meanwhile, the existing techniques come with their own set of controversies.

During 2000, Erol [30] presented the idea of a Turkish Delight, where Surgicel was used to wrap up diced cartilage, before it was used in rhinoplasty as an adjunct. This technique has been rejected by many studies because of its inability to fix the issue because of complete resorption by the three-month mark, where the process of using temporalis fascia wrapping began [1]. This method may be just 10 years old, and yet has found itself in the midst of a great debate. Some studies have histologically demonstrated that diced cartilage wrapped in fascia can remain stable for a period longer than nine months [31].

The diced cartilage histology shows the graft’s stability and viability four and a half years after it was injected [32]. An ingrowth of vascularised tissue with collagen deposition later occurred around the diced cartilage and helped achieve a solid fusion of the diced cartilage graft with connective tissue filling in the interstices. The viability of diced cartilage was compared with crushed or morselised cartilage graft in several animal studies. Diced cartilage has shown superior results with more viable chondrocytes compared with the other two methods of graft preparation [27,33,34].

Adopting any new technique when it comes to grafting will need answers to several key questions. This includes the merits and demerits of the technique in question, and if it will prove to be safe to implement in the longer term.

It is less harmful for the cartilage structure to be diced into smaller pieces through the use of sharp blades. It has been observed in many clinical and experimental studies that this maintains its viability over time [35]. If it is wrapped in good material, the fascia can enhance the dorsum; however, it is typically too voluminous to be used to correct minor issues. If used without a fascia to cover it, it can be added in smaller quantities during the procedure itself, where no tight pockets from the adjoining tissues can hinder its displacement [35].

Mincing or dicing cartilage into smaller pieces can be hard, and is often impossible, because they had to be small enough to be able to pass through the cannula. This is why conventional blades are not used, and instead powered devices or an otologic burr is used to do the job. However, the extent to which the chondrocytes are damaged during histological analysis might not support longer term survival [11].

The injectable fluid cartilage’s prep itself is a simple process that requires no special devices or facilities. It normally adds little interference to the lacuna or chondrocytes’ integrity, and can be applied through a 21-gauge needles or 1–2-mm diameter cannula with a blunt tip. Vigorously crushing a cartilage for the purpose of injecting it can only lead to the anatomical structure being destroyed. It can lead to a more unstable, and unpredictable, resorption.

Fluid cartilage has the advantage of getting injected into the mucosa or skin in closed space and allowing the surgeon to incrementally fix the deformity, and to the extent desired with specific analysis of the changes in the surface.

In conclusion, chondrocytes and fluid cartilage may be an interesting and possibly significant source of autologous tissue, which is ready to be utilised in therapeutic procedures, such as the regeneration or repair of nose tissue defects and irregularities of the nose. With the filler of the fluid cartilage, the septal cartilage tissue could present a new use in clinical terms, and allow for new strategies during the procedure.

The advantages of the cartilage fluid are that it fits exactly the desired shape moulding it, to distribute smoother and correctly the graft, in the same way as a filler, without undermining the nose flap, which is sometimes very dangerous because weaker tissues and altered anatomy planes can bring further tissue injuries; this allows avoidance of the displacement of the graft into death space. However, contrary to the filler, which remains fluid and is reabsorbed, the fluid cartilage solidifies in autologous tissue and survives for a lesser loss of chondrocytes. Considering that there are not surgical dissections, the nose appears untouched, without important brushing and swelling, and with a short recovery time.

The disadvantages are that it is time-consuming, compared with the traditional rhinofiller. Moreover, due to the loss of the cartilage in the dead space of the needles, it is not possible to push with saline, because as observed, they pass only the saline, without cartilage. Another disadvantage is the possible rate of resorption of volume that makes it necessary to repeat the procedure. We are not able to determine the percentage of cartilage survival. There were not overcorrections; rather, another procedure is considered if necessary.

Although this serves as a preliminary report, the findings are very promising when it comes to new therapeutic approaches for rhinoplasty. Furthermore, using experimental protocols in actual clinical care and employing the substantial regenerative capacity of the human tissue can create new approaches.

Recently, the authors also started used hyaluronic acid (HA) to rehydrate but also better aggregate the cartilage (replacing the saline solution used instead in this study), and they started to use the fluid cartilage as scaffold for Nano fat gel. Nano fat gel was derived from the harvesting of micro fat in the superficial layer of adipose tissue where there is more yield of adipose-derived stem cells (ADSCs) [36]. The idea to use Nano fat gel in combination with fluid cartilage aimed to stimulate angiogenesis through ADSC cells and also improve the softness of the soft tissue treated.

## 5. Conclusions

Often after rhinoplasty, the minor irregularities are generally given little thought or attention, but they must be treated as precisely as possible to achieve the perfect profile. Our information obviously highlights the constructive effects of fluid cartilage filling on minimal irregularities of the nasal surface and the absence of major side effects. Fluid cartilage may fill in as a safe and effective treatment alternative to hyaluronic acid or traditional cartilage graft; more broad controlled examinations are required.

## Figures and Tables

**Table 1:**
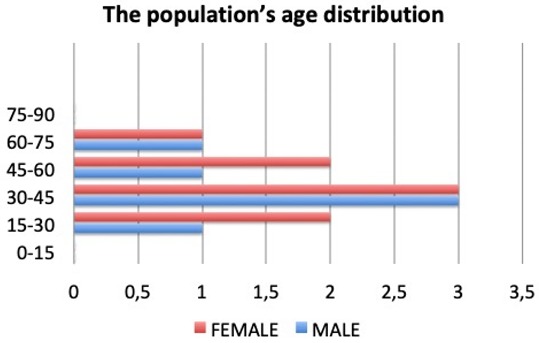
**The population’s age distribution**. Relationship between age (axis y) and number of patients treated (axis x).

## Supplementary Materials

The following are available online at https://www.mdpi.com/1996-1944/12/7/1062/s1, Video S1: fluid cartilage as a filler.

## Abbreviations

PRP	Platelet-Rich-Plasma
EC	European Parliament
CAT	Committe for Advanced treatments
GMP	Good Manufacturing Practices
GCP	Good Clinical Practices
SPSS	Statistical Package for the Social Sciences
CD	Cluster of differentiation
NaCl	Saline solution
MW	Molecular weight
PCR-	Negative control
HCs	Human Condrocytes
SD	Standard Deviation
